# Mangotoxin production of *Pseudomonas syringae* pv. syringae is regulated by MgoA

**DOI:** 10.1186/1471-2180-14-46

**Published:** 2014-02-21

**Authors:** Víctor J Carrión, Menno van der Voort, Eva Arrebola, José A Gutiérrez-Barranquero, Antonio de Vicente, Jos M Raaijmakers, Francisco M Cazorla

**Affiliations:** 1 Departamento de Microbiología, Facultad de Ciencias, Instituto de Hortofruticultura Subtropical y Mediterránea “La Mayora”-Universidad de Málaga-Consejo Superior de Investigaciones Científicas (IHSM-UMA-CSIC), Universidad de Málaga, Campus de Teatinos, 29071 Málaga, Spain; 2 Instituto de Hortofruticultura Subtropical y Mediterránea “La Mayora”-Universidad de Málaga-Consejo Superior de Investigaciones Científicas (IHSM-UMA-CSIC), Estación Experimental La Mayora, Algarrobo-Costa, 29750 Málaga, Spain; 3 Laboratory of Phytopathology, Wageningen University, Wageningen, 6708 PB The Netherlands; 4 Department of Microbial Ecology, The Netherlands Institute of Ecology (NIOO-KNAW), Wageningen, The Netherlands; 5 BIOMERIT Research Centre, School of Microbiology, University College Cork, National University of Ireland, Cork, Ireland

**Keywords:** Antimetabolite toxin, *mgo* operon, GacS/GacA, Plant-microbe interaction

## Abstract

**Background:**

The antimetabolite mangotoxin is a key factor in virulence of *Pseudomonas syringae* pv. syringae strains which cause apical necrosis of mango trees. Previous studies showed that mangotoxin biosynthesis is governed by the *mbo* operon. Random mutagenesis led to the identification of two other gene clusters that affect mangotoxin biosynthesis. These are the *gacS/gacA* genes and *mgo* operon which harbors the four genes *mgoBCAD*.

**Results:**

The current study shows that disruption of the nonribosomal peptide synthetase (NRPS) gene *mgoA* resulted in loss of mangotoxin production and reduced virulence on tomato leaves. Transcriptional analyses by qPCR and promoter reporter fusions revealed that *mbo* expression is regulated by both *gacS/gacA* and *mgo* genes*.* Also, expression of the *mgo* operon was shown to be regulated by *gacS/gacA*. Heterologous expression under the native promoter of the *mbo* operon resulted in mangotoxin production in non-producing *P. syringae* strains, but not in other *Pseudomonas* species. Also introduction of the *mbo* and *mgo* operons in nonproducing *P. protegens* Pf-5 did not confer mangotoxin production but did enhance transcription of the *mbo* promoter.

**Conclusions:**

From the data obtained in this study, we conclude that both *mbo* and *mgo* operons are under the control of the *gacS/gacA* two-component system and that the MgoA product acts as a positive regulator of mangotoxin biosynthesis.

## Background

*Pseudomonas syringae* is one of the most ubiquitous plant pathogens, causing various economically important diseases [1]. The present study focuses on *P. syringae* pv. syringae UMAF0158 (CECT 7752) which causes apical necrosis of mango [2,3]. The antimetabolite mangotoxin is a key virulence factor of strain UMAF0158 [4,5]. This toxin is produced in the early exponential growth phase and inhibits ornithine *N*-acetyl transferase, a key enzyme belonging to the ornithine/arginine biosynthetic pathway [2].

Random mini-Tn*5* mutagenesis followed by cloning, sequencing and heterologous expression recently led to the identification of the gene cluster that governs mangotoxin biosynthesis [6]. The *mbo* operon (**m**angotoxin **b**iosynthetic **o**peron) is composed of six genes, *mboABCDEF*. Disruption of each of these genes resulted in mangotoxin deficient mutants and constitutive expression of the *mbo* operon in non-mangotoxin producing *P. syringae* strains conferred mangotoxin production [6]. Screening of the random mutant library also led to the identification of several other genes that may be involved in the regulation of mangotoxin biosynthesis [4]. These included the *gacS/gacA* genes and the so-called **m**angotoxin **g**enerating **o**peron *mgo*[6,7].

The GacS/GacA two-component regulatory system is highly conserved in Gram-negative bacteria and is involved in a variety of functions, including pathogenicity [8], quorum sensing [9,10], secondary metabolite production [11-14] and biofilm formation [15-17]. In *Pseudomonas syringae,* the GacS/GacA two-component system regulates the production of the phytotoxins syringomycin and syringopeptin [18-20], tabtoxin [21,22] and phaseolotoxin [23]. In *P. syringae* pv. tomato DC3000, GacS/GacA regulate the *hrpR*, *hrpS*, and *hrpL* genes, which are required for the activation of the Hrp type III secretion and effector genes [24,25]. However, in *P. syringae* pv. syringae B728a, GacA appears not to be required for *hrp* gene expression [25].

The *mgo* operon is composed of four genes, *mgoBCAD*[4,7]. Mutants in each gene belonging to the *mgo* operon showed an alteration (*mgoB* mutant) or lack of mangotoxin production (*mgoC*, *mgoA* and *mgoD* mutants). These genes encode for different hypothetical proteins with predicted domains for a haem oxygenase (MgoB), a *p*-aminobenzoate *N*-oxygenase (MgoC), a nonribosomal peptide synthetase (MgoA), and a polyketide cyclase/dehydrase or lipid transporter (MgoD) [4,7]. The predicted amino acid sequence of MgoA suggests only one amino acid activation module and 14 conserved domains, including aminoacyl adenylation, condensation, thiolation, and additional reduction domains [4]. Genes homologous to the *mgo* operon have been found in the genomes of most *Pseudomonas* spp., with the exception of *P. protegens* Pf-5 and CHAO [26,27]. Recent studies on the *pvf* gene cluster in *P. entomophila*, a homologue of the *mgo* operon, suggested that it affects virulence [28]. Almost all the fluorescent *Pseudomonas* spp. lack the *mbo* operon [29,30], but the *mgo* operon is conserved in all of them (except *P. protegens* Pf-5) [4,7,26-28]. To date, however, the functions of *mgo* operon are yet unknown.

The overall objective of this study was to get insight into the role of the *mgo* operon in regulation of mangotoxin production in *P. syringae* pv. syringae UMAF0158 and unravel the interplay between *mgo*, *mbo* and the *gacS/gacA* two-component regulatory system.

## Methods

### Bacterial strains and culture conditions

The wild type strain *P. syringae* pv. syringae UMAF0158 (CECT 7752) and the collection of selected derivative mutants used in this study (Table 1) were grown on *Pseudomonas* agar F (Difco) plates, in liquid King’s medium B (KMB) [31] or in *Pseudomonas* minimal medium (PMS) [32] at 28°C. *Escherichia coli* strain DH5α was used as a host for plasmid complementation experiments. It was routinely grown on Luria-Bertani (LB) plates or in LB broth at 37°C. Antibiotics for selection of *P. syringae* pv. syringae UMAF0158 and *E. coli* derivatives were ampicillin (100 mg L^-1^), kanamycin (50 mg L^-1^), gentamycin (30 mg L^-1^) or tetracycline (25 mg L^-1^).

**Table 1:** Bacterial strains and plasmids used in this study

**Strain or plasmid**	**Relevant characteristics**	**Reference/source**
**Strains**		
*E. coli*		
DH5α	*E. coli [F’ Φ80lacZ ∆M15 ∆(lacZYA-argF)U169 deoR recA endA1 hsdR17 (rK-mK+)phoA supE44 lambda- thi-1]*	[33]
CECT831	Indicator strain for mangotoxin production	CECT^a^
*P. syringae* pv. syringae		
B728a	Complete genome, non-mangotoxin producer	[34]
UMAF0158	Wild type, isolated from mango, mangotoxin producer, Nf^r^	[2]
*mboA*^**-**^	Derivative mutant of UMAF0158 by insertion in *mboA*, Km^r^, Nf^r^ (named *mbo*A^-^)	[6]
*∆mgoA*	Derivative mutant of UMAF0158 by deletion of *mgoA*, Nf^r^ (named *∆mgo*A)	[7]
2βB7	miniTn*5* mutant of UMAF0158 in *gacA* defective in mangotoxin, Km^r^, Nf^r^ (named *gac*A^-^)	[4]
3αE10	miniTn*5* mutant of UMAF0158 in *gacS* defective in mangotoxin, Km^r^, Nf^r^(named *gac*S^-^)	[2]
3γH1	miniTn*5* mutant of UMAF0158, defective in mangotoxin production, Km^r^, Nf^r^	[2]
4βA2	miniTn*5* mutant of UMAF0158, defective in mangotoxin production, Km^r^, Nf^r^	[2]
5αC5	miniTn*5* mutant of UMAF0158, defective in mangotoxin production, Km^r^, Nf^r^	[2]
6γF6	miniTn*5* mutant of UMAF0158, defective in mangotoxin production, Km^r^, Nf^r^	[2]
*P. protegens* Pf-5	Non mangotoxin producer, *mbo* and *mgo* operon absent	[35]
**Plasmids**		
pBBR1MCS-5	4.7 kb broad-host-range cloning vector, Gm^r^	[36]
pGEM-T	3.0 kb cloning vector, Ap^r^	Invitrogen
pGEM-T*BCAD*	*mgo*BCAD cloned in pGEM-T, Ap^r^	This study
pLac-*mgoBCAD*	*mgo*BCAD cloned in pBBR1MCS-5 downstream the *lacZ* promoter in the vector, *mgo* operon expression under its own and P_*LAC*_ promoter, Gm^r^	This study
pLac-*mboABCDEF*	*mboABCDEF* cloned in pBBR1MCS-5 downstream the *lacZ* promoter in the vector, *mbo* operon expression under its own and P_*LAC*_ promoter, Gm^r^	[6]
pLac-*mboFEDCBA*	*mboABCDEF* cloned in pBBR1MCS-5 in the opposite direction than the *lacZ* promoter in the vector, *mbo* operon expression under its own promoter, Gm^r^	[6]
pMP220	Promoter-probe vector containing a promoterless LacZ gene, Tet^r^	[37]
pMP-*mboABCDEF*	*mboABCDEF* cloned in promoter-probe vector containing a promoterless LacZ gene, *mbo* operon expression under its own promoter, Tet^r^	This study
pMP::P_*mboI*_	pMP220 vector containing the *mbo* operon promoter, Tet^r^	[6]

^a^CECT: Spanish Type Culture Collection, Spain.

### Mangotoxin production assay

Antimetabolite toxin production was assayed by the indicator technique previously described [32]. Briefly, a double layer of the indicator microorganism *E. coli* CECT 831 was prepared; after solidification, the *P. syringae* pv. syringae strains to be tested were stab-inoculated. The plates were initially incubated at 22°C for 24 h, and then at 37°C for an additional 24 h [2]. To evaluate mangotoxin activity, the same plate bioassay was carried out with the addition of 100 μl of a 6 mM solution of *N*-acetyl-ornithine or L-ornithine to the double layer of *E. coli*[2]. To determine growth characteristics of representative strains, the wild type mangotoxin-producing *P. syringae* pv. syringae UMAF0158 and derivatives mutants in *mboA, mgoA* and *gacA* genes were used to obtain initial cultures in 10 ml of LB broth. The bacterial strains were grown during 24 h at 28°C to prepare an optimal bacterial inoculum with an optical density of 0.8 at 600 nm (approximately 10^9^ cfu ml^-1^). One ml from these bacterial inocula was used to inoculate 100 ml of PMS broth. The cultures were incubated at 22°C under orbital shaking at 150 rpm until the stationary phase. Samples were collected every 6 or 12 h to monitor the bacterial growth. Bacterial cfu per sample were determined by 10-fold serial dilutions on KMB plates. At the same time, the mangotoxin production assessment was performed by a cell-free filtrate dilution sequence at 50%. The mangotoxin production is measured using arbitrary units, which can be defined as the relative toxic volume of cell free filtrates of liquid cultures, which produces an inhibition halo of 18 mm in diameter under standard assay conditions [2]. The methodology presented a detection threshold of 0.5 toxic units, due to the diameter of the wells where the cell-free filtrate were deposited (9 mm).

### Complementation experiments

DNA fragments of approximately 7 kb containing the *mgo* and *mbo* operons, including the promoter and terminator regions, were obtained by PCR using specific primers (Additional file 1: Table S1) and high fidelity polymerase (Phusion DNA polymerase, Finnzymes). The PCR amplification products were cloned in pGEM-T Easy (Promega), and the plasmids obtained were digested with *Xba*I for the *mgo* operon and with *EcoR*I and *Pst*I for the *mbo* operon. After the digestion, both operons fragment were obtained from gel with the NucleoSpin kit (GE Healthcare) and cloned into the correspondent shuttle vectors, pBBR1MCS-5 [36] for the *mgo* operon and pMP220 [37] for the *mbo* operon, which were digested, dephosphorylated (shrimp alkaline phosphatase; Promega), and purified with the NucleoSpin kit according to the manufacturer’s instructions. *E. coli* DH5α was transformed with the plasmids obtained, by heat shock transformation [38], and transformed colonies were selected on LB agar plates supplemented with gentamicin (30 mg L^-1^) in the case of pBBR1MCS-5 and tetracycline (25 mg L^-1^) for pMP220. Plasmids with the *mgo* and *mbo* operon cloned were obtained (Table 1). Correct integration and orientation of the fragments was verified by PCR and restriction analysis of isolated plasmids (data not shown). The pLac-*mgoBCAD* construct was subsequently electroporated into the *mboA, mgoA* and *gacA* mutants, and the wild-type strains *P. syringae* pv. syringae UMAF0158 and *P. protegens* Pf-5. The pMP-*mboABCDEF* construct was transformed in *P. protegens* Pf-5 which previously contain the pLac-*mgoBCAD*, therefore this bacteria finally harbored both operons, the *mgo* and *mbo* operon. Transformed cells were selected on KMB agar supplemented with correspondent antibiotics. The presence of the different plasmids was confirmed by PCR analysis with specific primers for pBBR1MCS-5 and pMP220 and plasmid profiling.

### Virulence evaluation

The virulence of different mangotoxin producing or non-producing *P. syringae* pv. syringae strains were analyzed in detached tomato leaflets (*Solanum lycopersicum* Mill.) cv. Hellfrucht Frühstamm maintained *in vitro* using Murashige and Skoog medium (MS, Sigma-Aldrich) [4,5]. Bacterial suspensions from exponentially growing cultures were adjusted to 10^8^ cfu ml^-1^. The leaflets were inoculated by placing six 10 μl drops of the bacterial suspension on six different points on the same leaflet. Inoculations were then carried out by piercing through the droplets with a sterile entomological pin. The leaflets were maintained in MS media at 22°C and a 16:8-h light: dark photoperiod. Six tomato leaflets were used to evaluate each strain. Detached leaflets only inoculated with sterile distilled water were included in all experiments as a control. These experiments were repeated three times. The development of necrotic symptoms at the inoculation points (n = 108) was determined after 10-day. The severity symptoms were evaluated by the analysis of the total necrotic area per leaflet induced by the inoculated strains after 10 days of incubation. For severity measurement, the necrotic areas of the inoculation points were digitally analyzed on the six leaflets, using the computer image software VISILOG 5.0 (Noesis Vision Inc.). At the same time, two inoculated leaflets were used to estimate the daily development of the total bacterial population. For that purpose, whole tomato leaflets were homogenized in sterile water and bacterial counts were determined plating by 10-fold serial dilutions on KMB plates. Bacterial growth inside the plant tissue was recorded after H_2_O_2_ leaf surface disinfection. Colony counts growth based on the typical morphology of *P. syringae* pv. syringae UMAF0158 were recorded after incubation at 28°C for 48 h.

### Transcriptional analysis

From PMS cultures described above, cells from 2 ml cultures were collected and spun down at 12,000 rpm (1 min) from the wild type strain and the derivative mutants in *gacA* and *mgoA*. The cells were frozen in liquid N_2_ and stored at -80°C. For the RNA isolations and cDNA synthesis, three biological replicates were used for each time point. For the transcriptional analyses, RNA was isolated from the frozen bacterial cells with Trizol reagent (Invitrogen), followed by DNase I (GE Healthcare) treatment. One μg of RNA was used for cDNA synthesis with Superscript III (Invitrogen) according to the manufacturer’s protocol. For the real-time quantitative PCR (Q-PCR), conducted with the 7300SDS system from Applied Biosystems, the SYBR Green Core kit (Eurogentec) with a final concentration of 3.5 mM MgCl_2_ was used according to the manufacturer’s protocol. The concentration of the primers was optimized (400 nM final concentration for all of them), and a dissociation curve was performed to check the specificity of the primers. The primers used for the Q-PCR are listed in Additional file 1: Table S1. To correct for small differences in template concentration, *rpoD* was used as the reference housekeeping gene. The cycle in which the SYBR green fluorescence crossed a manually set cycle threshold (*C*_*T*_) was used to determine transcript levels. For each gene, the threshold was fixed based on the exponential segment of the PCR curve. The *C*_*T*_ value of *mboA* was corrected for the housekeeping gene *rpoD* as follows: ∆*C*_*T*_ = *C*_*T*_ (*mboA*) - *C*_*T*_ (*rpoD*); the same formula was used for the other genes studied. The relative quantification (RQ) values were calculated by the following formula: RQ = 2^− [*ΔCT*(mutant) − *ΔCT*(wild type)]^[39,40]. Q-PCR analysis was performed in duplicate (technical replicates) on three independent RNA isolations (biological replicates).

### β-galactosidase assays

To study the *mbo* operon expression in different genetic backgrounds, the *mbo* operon promoter (P_*mboI*_) cloned into pMP220 [19] as previously described [6] was used. The derivative mutants in *mgoA, gacA* and *gacS* genes were transformed with plasmid pMP::P_*mboI*_ which contains the P_*mboI*_. The plasmid pLac-*mgoBCAD* (harboring the *mgo* operon) was also used to complement the *mgoA*, *gacA* and *gacS* mutants and finally the β-galactosidase activity of P_*mboI*_ was measured. In order to evaluate the effect of the *mgo* operon on the activity of P_*mboI*_, *P. protegens* Pf-5 was used due to the absence of the two operons in its genome. First, *P. protegens* Pf-5 was transformed with the pMP::P_*mboI*_ and the promoter activity was measured, and secondly to measure the effect on the *mbo* operon transcription, this strain containing the plasmid pMP::P_*mboI*_, was also transformed with the plasmid pLac-*mgoBCAD* (*mgo* operon under pLac regulation). As a negative control the β-galactosidase activity was measured for the wild type strain *P. syringae* pv. syringae UMAF0158 and each strain used in this assay, transformed with empty vector pMP220. β-galactosidase activities were quantified by the Miller method [41]. Briefly, an overnight culture obtained as previously described in growth curve and toxins assay section were prepared. The samples were collected at 18 h, and the cells were harvested and suspended in assay buffer to eliminate any error in the detection of β-galactosidase activity due to the effects of different carbon sources present in the growth medium. The results presented are from three separate experiments, each conducted in triplicate.

### Phylogeny of the *mgoA* gene

In order to identify the presence of the *mgoA* gene in the different genomes of *Pseudomonas* strains, the *mgoA* gene from *P. syringae* pv. syringae UMAF0158 was used in BLASTP [42] comparisons with whole genome sequences of *Pseudomonas* spp. available in the databases. Once the amino acid sequences of all the orthologous *mgoA* genes were obtained, the putative adenylation domains were identified using the PKS/NRPS Analysis Web-site (http://nrps.igs.umaryland.edu/nrps) [43]. Other adenylation domains of which the activated amino acid is already known were obtained from the database and from De Bruijn met al. [44]. Two phylogenetic analyses were done, the first was using the adenylation domain of all the NRPSs (328 residues) and the second was using the almost entire sequence of MgoA (1015 residues). Amino acid sequences were aligned with Muscle (MEGA5 software) and determination of the optimal amino acid substitution model and phylogenetic tree construction were done using MEGA5 software [45]. Neighbor-joining, maximum parsimony and maximum-likelihood phylogenetic trees of the individual gene sequences were generated in MEGA5 by using the optimal model parameters and the option of complete deletion to eliminate positions containing gaps. Confidence levels for the branching points were determined using 1,000 bootstrap replicates.

### Bioinformatics and statistical analysis

Searches for sequence similarity in the NCBI databases were carried out using BLAST algorithms [42]. Genome and nucleotide sequences were visualized and manipulated using the Artemis genome browser [46] and compared using ACT [47] in combination with WebACT [48]. The statistical analysis of incidence was performed by SAS9.2 software (SAS Institute Inc.) by Enterprise Guide 4.2 using generalized linear model analysis. The β-galactosidase and the necrotic area data were statistically analyzed using an analysis of variance, followed by Fisher’s least significant difference test (p = 0.05), and for β-galactosidase activity on *P. protegens* Pf5, a Student’s *t*-test was carried out (p = 0.05), using the IBM.SSPS 19 software (IBM® Company).

## Results

### Involvement of *mbo* genes in mangotoxin production and virulence in *P. syringae* pv. syringae UMAF0158

**Figure 1:**
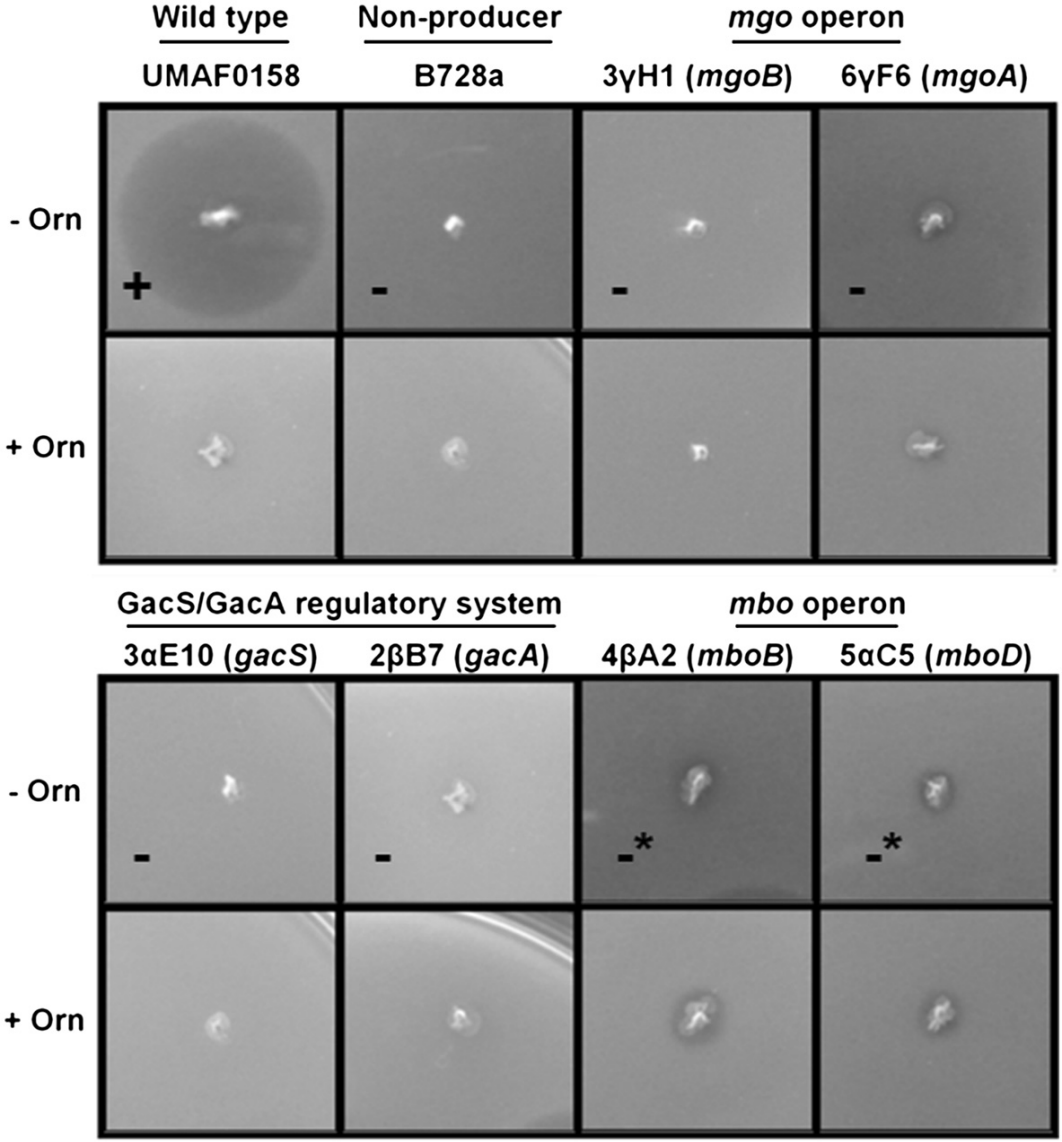
**Mangotoxin production by random miniTn*5*insertional mutants.** Three pairs of mutants in different genes of the mbo and mgo operon, and in the gacS/gacA two-component regulatory system, obtained in previous works and tested for mangotoxin production. The corresponding disrupted gene is detailed in brackets. The P. syringae pv. syringae strains UMAF0158 (mangoxin-producing wilt-type strain) and B728a (nonproducing) were used as references. Mangotoxin production was evaluated using PMS minimal medium supplemented or not with ornithine. The results are indicated as follows: - absence of inhibition halo, + presence of inhibition halo, -* slight toxicity which was not reverted by addition of ornithine. Toxic activity reverted in presence of ornithine denotes the production of mangotoxin.

Six mangotoxin deficient mutants of *P. syringae* pv. syringae UMAF0158, were previously obtained and characterized for mangotoxin production (Table 1 and Figure 1). Mangotoxin characterization showed that although these mutants did not show mangotoxin production, a slight production of a yet unknown antimicrobial compound was observed for mutants 4βA2 (*mboB*) and 5αC5 (*mboD*) (Figure 1). For two mutants (3γH1 and 6γF6), the Tn*5* insertion was located in *mgoC* and *mgoA* respectively. Two other non-mangotoxin producing mutants were disrupted in the genes encoding the GacS/GacA two-component regulatory system (3αE10 and 2βB7 respectively). Growth of the *mgoA* mutant was shown to be similar to that of the wild type strain, with cell densities of up to 10^11^ cfu ml^-1^ in liquid medium after 108 h of growth at 22ºC (Additional file 2: Figure S1A). In contrast, the *gacA* mutant presented an altered growth, with cell densities in the stationary phase reaching only 10^9^ cfu ml^-1^ (Additional file 2: Figure S1A). The dynamics of the mangotoxin production in relation to bacterial growth was followed during four days of incubation. Mangotoxin production was detectable after 24 h of growth, increased up to 1.4 toxic units (T.U.), then reduced slightly upon entry of the stationary phase and then stabilized (Additional file 2: Figure S1B).

In order to know if the virulence of the derivative mutants *mboA*- and *mgoA* was reduced in comparison with the wild type strain, detached tomato leaflets were artificially inoculated. Artificial inoculation experiments using detached tomato leaflets [4] showed that bacterial growth inside the tomato leaflets of the *mboA*^*-*^ and Δ*mgoA* mutants as well as their complemented derivatives followed similar dynamics (Additional file 3: Figure S2A). When inoculations were performed, development of necrotic lesions was observed on the leaf. Disease severity, represented by the necrotic area, showed that both mangotoxin defective mutants were less virulent than the wild type UMAF0158 (Additional file 3: Figure S2B and S2C). When derivative strains were complemented with the *mboA* and *mgoA* genes disease severity increased but complementation did not fully restore virulence to wild type level (Additional file 3: Figure S2B and S2C).

### Mangotoxin production and transcriptional regulation in the *gacA* and *mgoA* mutant

**Figure 2:**
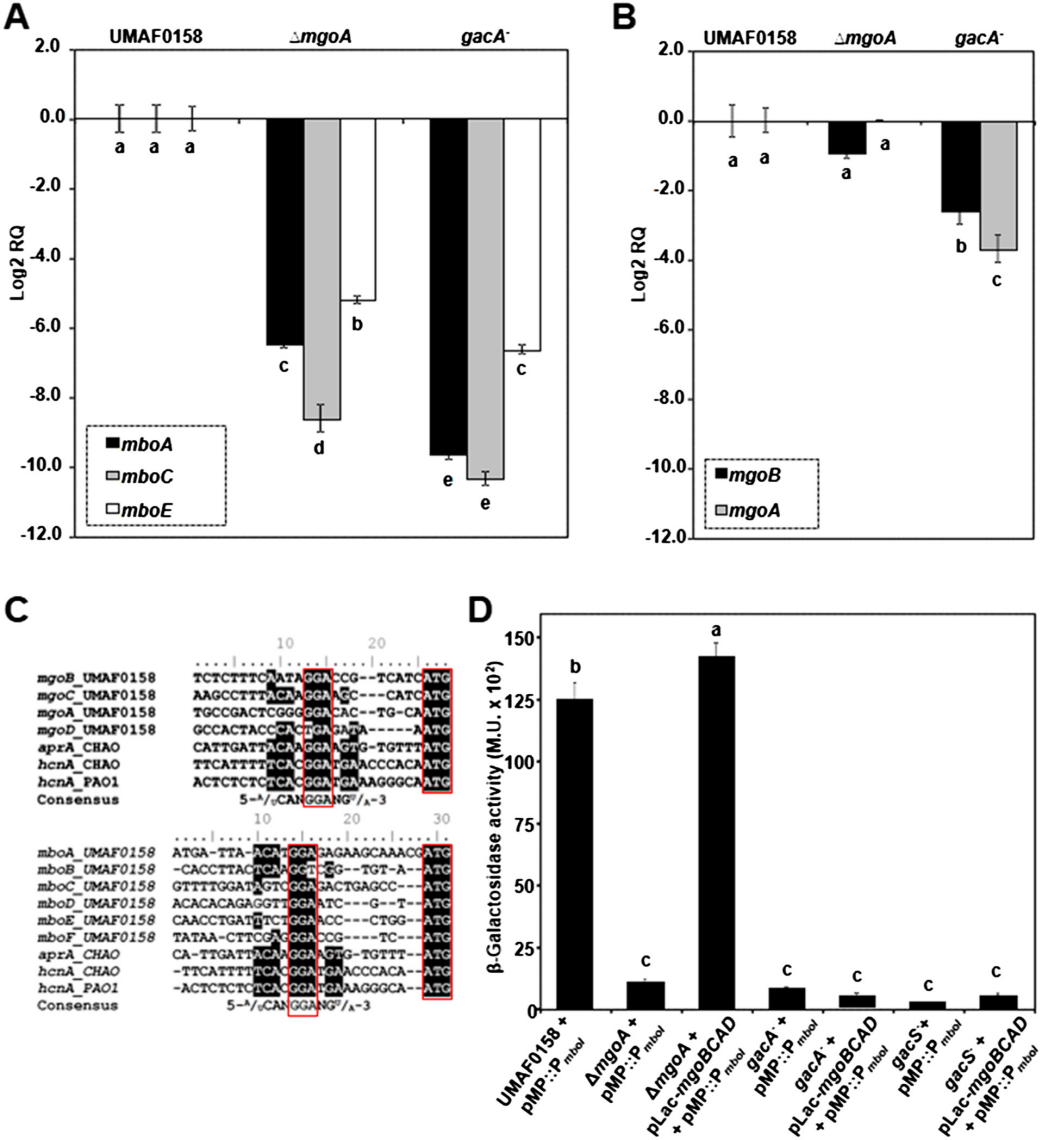
**Transcriptional analysis and*mbo*operon promoter activity.***mboA*, *mboC* and *mboE***(A)**, belonging to the *mbo* operon and *mgoB* and *mgoA***(B)**, belonging to the *mgo* operon transcript levels in the wild type strain *P. syringae* pv. syringae UMAF0158 and *mgoA* and *gacA* mutants. **(C)** Comparison of the described consensus motif (5′-CANGGANG-3′) for *P. fluorescens*[49-51]: The search was done in front of each start codon of the *mgo* and *mbo* genes. **(D)** β-galactosidase activity of the *mbo* operon promoter in the wild-type strain UMAF0158 and *mgoA*, *gacS* and *gacA* mutants. These strains were transformed with the *mbo* operon promoter named pMP::P_*mboI*_ and the empty promoter-probe vector pMP220 was used as a control. The different mutants were also transformed with the vector pLac-*mgoBCAD*. Log2RQ represents the expression levels of the studied genes by relative quantification scores. Values below 0 indicates lower expression than the housekeeping gene used for normalization of data. The results are average of three independent experiments performed in triplicate. Error bars indicate standard deviation. Data were analysed for significance using an arcsine square root transformation with analysis of variance followed by Fisher’s least significant difference test (P = 0.05). Values of bars with different letter designations represent a statistically significant difference.

To study the role of *mgoA* and *gacA* in mangotoxin biosynthesis, transcription of the *mboACE* and *mgoBA* genes was analyzed for the wild type strain, and for the *mgoA* and *gacA* derivative mutants. Time course experiments showed that the *mbo* genes in the wild type are expressed at the highest level after 12 to 24 h (Additional file 4: Figure S3). Therefore all comparisons between wild type and mutants were performed at 18 h of growth. Transcript levels of the *mboACE* genes after 18 h of growth were significantly lower in the *gacA* and the *mgoA* mutants than in the wild type (Figure 2A). Also the transcript levels of *mgoB* and *mgoA* were significantly lower in the *gacA* mutant (Figure 2B). The *mgoA* mutation did not affect transcription of *gacS/gacA* (data not shown). Also *mboA*, *mboC*, or *mboE* mutations did not significantly affect transcription of *gacS/gacA* or *mgoA* (data not shown). These results indicate that the GacS/GacA two-component regulatory system affects transcription of both the *mbo* and *mgo* genes and that the product of the *mgo* operon influences transcription of the *mbo* genes. To further study if the GacS/GacA two-component regulatory system could regulate the *mgo* and *mbo* genes via RNA repressor binding proteins [49-51], the upstream regions of the *mgo* and *mbo* genes were inspected for the presence of the described consensus motif (5′-CANGGANG-3′) previously described in *P. protegens* CHAO [49]. This motif allows the binding of the repressor to the RNA, and these repressor proteins can be removed by Gac/Rsm. The complete consensus sequence was not detected upstream of any of the *mbo*/*mgo* genes (Figure 2C). However, consensus GGA motifs for binding of the RNA binding proteins [49-51] were detected upstream of the *mbo* and *mgo* operons (Figure 2C). It must be taken into account that the described consensus sequence is from *P. protegens*[49], and nothing is known yet about the recognition site of RNA binding proteins in *P. syringae*.

As the transcription of the *mgo* operon was substantially lower in the *gacA* mutant (Figure 2B), we subsequently tested whether introduction of extra copies of the *mgo* operon in the *gacS* or *gacA* mutant could restore mangotoxin production. When the *mgo* operon was introduced in the *mgoA* mutant mangotoxin production was restored, which was not the case for the *mboA*, *gacA* and *gacS* mutants (Table 2).

**Table 2:** **Toxic activity of** ***P. syringae*** **pv syringae UMAF0158 mutants and** ***mgo*** **operon complemented strains**

**Strains**	***E. coli*** **inhibition assay**		**Mangotoxin production**
	**PMS**	**PMS + ornithine**	
***Wild type strain and derivative mutants***			
UMAF0158	+	-	Yes
*mboA*^-^	-*	-*	No
Δ*mgoA*	-	-	No
*gacA*^-^	-	-	No
*gacS*^-^	-	-	No
***Transformed with empty vector***			
UMAF0158	+	-	Yes
*mboA*^-^	-*	-*	No
Δ*mgoA*	-	-	No
*gacA*^-^	-	-	No
*gacS*^-^	-	-	No
***Transformed with pLac-mgoBCAD***			
UMAF0158	++	-	Yes
*mboA*^-^	-*	-*	No
Δ*mgoA*	++	-	Yes
*gacA*^-^	-	-	No
*gacS*^-^	-	-	No

The results are indicated as follows: - absence of inhibition halo, + inhibition halo between 5-10 mm, ++ inhibition halo bigger 10 mm, -* slight toxicity which did not revert in presence of ornithine. Toxic activity, which reverts in the presence of ornithine, denotes the production of mangotoxin.

### The *mgo* operon is a positive regulator of *mbo* operon transcription

To further elucidate the role of the *mgo* operon in the regulation of mangotoxin biosynthesis, expression assays were carried out using a plasmid reporter construction consisting of the *mbo* operon promoter fused to a promoterless *lacZ* gene. When the plasmid reporter was transferred into the wild type strain, high levels of β-galactosidase activity were found, whereas for the *mgoA, gacA* and *gacS* mutants this activity was substantially lower (Figure 2D). For the *mgoA* mutant, complementation with the *mgo* operon restored β-galactosidase activity to similar levels as in the wild type strain (Figure 2D). In contrast, no restoration of the β-galactosidase activity was found when the *mgo* operon was introduced in the *gacS/gacA*, confirming results described above (Table 2).

### MgoA phylogeny and mangotoxin production in other strains

**Figure 3:**
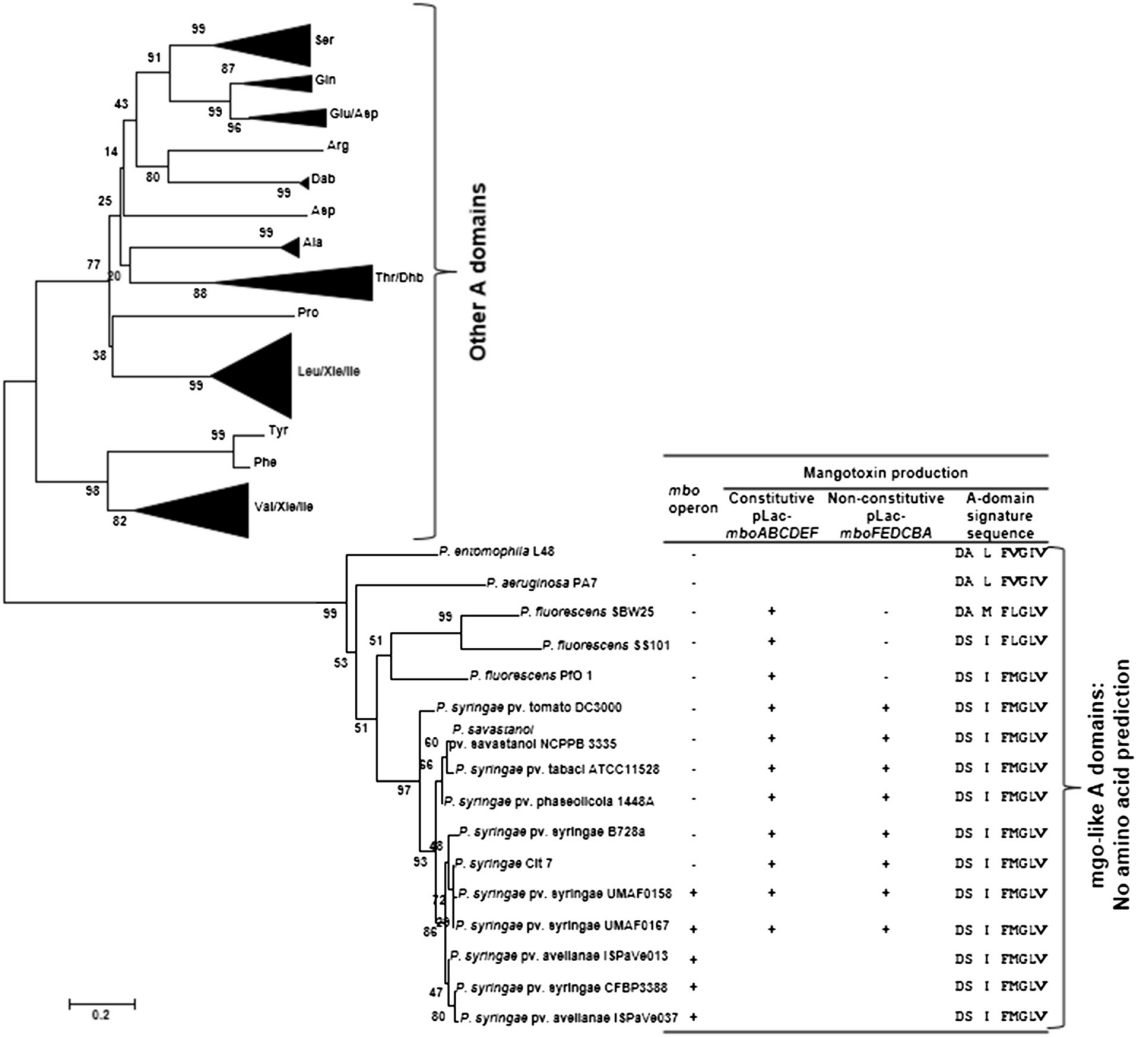
**Phylogeny of the MgoA adenylation domain.** Neighbor-joining tree, constructed with MEGA5 using the adenylation domains extracted from nonribosomal peptide synthetases involved in syringomycin, syringopeptin, massetolide A, arthrofactin synthesis and mangotoxin biosynthesis (MgoA). The presence (+) or absence (-) of the *mbo* operon is shown in the phylogenetic tree. The boxes indicate the different groups of *Pseudomonas* species which are able to produce mangotoxin when were transformed with pLac-*mboABCDEF* (*mbo* operon under its own and P_*LAC*_ promoter expression) or pLac-*mboFEDCBA* (*mbo* operon under its own promoter expression). Also is indicated the signature sequence of the adenylation domains in each strain. The evolutionary history was inferred using the Neighbor-Joining method [52]. The evolutionary distances were computed using the JTT matrix-based method [53] and are in the units of the number of amino acid substitutions per site. The variation rate among sites was modelled with a gamma distribution. The analysis involved 126 amino acid sequences. There were a total of 328 positions in the final dataset. Evolutionary analyses were conducted in MEGA5 [45]. Bootstrap values (1,000 repetitions) are shown on branches.

The amino acid sequence of a typical non-ribosomal peptide synthetase (NRPS) displays an adenylation (A) domain responsible for recognition and subsequent activation of an amino acid substrate. It also contains the typical thiolation (T) and condensation (C) domains. Finally, the thioesterase (TE) domain releases the final molecule from the NRPS assembly line. Based on the specific signature sequences described previously for A domains, analysis of MgoA did not allow prediction of the amino acid to be activated. Therefore, a phylogenetic analysis was performed with multiple A domains from NRPSs of which activated amino acids are known and with MgoA from other *Pseudomonas* species (Figure 3 and Additional file 5: Figure S4). The results showed that the A domains from the different MgoA orthologues grouped in the same cluster, separate from other A domains for which the activated amino acid residue is known (Figure 3).

To determine if *mgoA* present in other *Pseudomonas* species can regulate the *mbo* operon, reporter constructs pLac-*mboABCDEF* (*mbo* operon under its own and under pLac promoter expression) and pLac-*mboFEDCBA* (*mbo* operon only under its own promoter expression) were used. Firstly, only specific *P. syringae* pathovars harbor the *mbo* operon, and almost all strains from these pathovars produce mangotoxin [29], with or without the introduction of the *mbo* operon containing plasmids (Figure 3). Our results showed that other *P. syringae* pathovars, that do not contain the *mbo* operon, are all able to produce mangotoxin when they were transformed with pLac-*mboABCDEF* and pLac-*mboFEDCBA* (Figure 3). When different *P. fluorescens* strains were transformed with either vector, they only produced mangotoxin when the *mbo* operon was expressed constitutively but not when they were transformed with the *mbo* operon with its native promoter (Figure 3).

**Figure 4:**
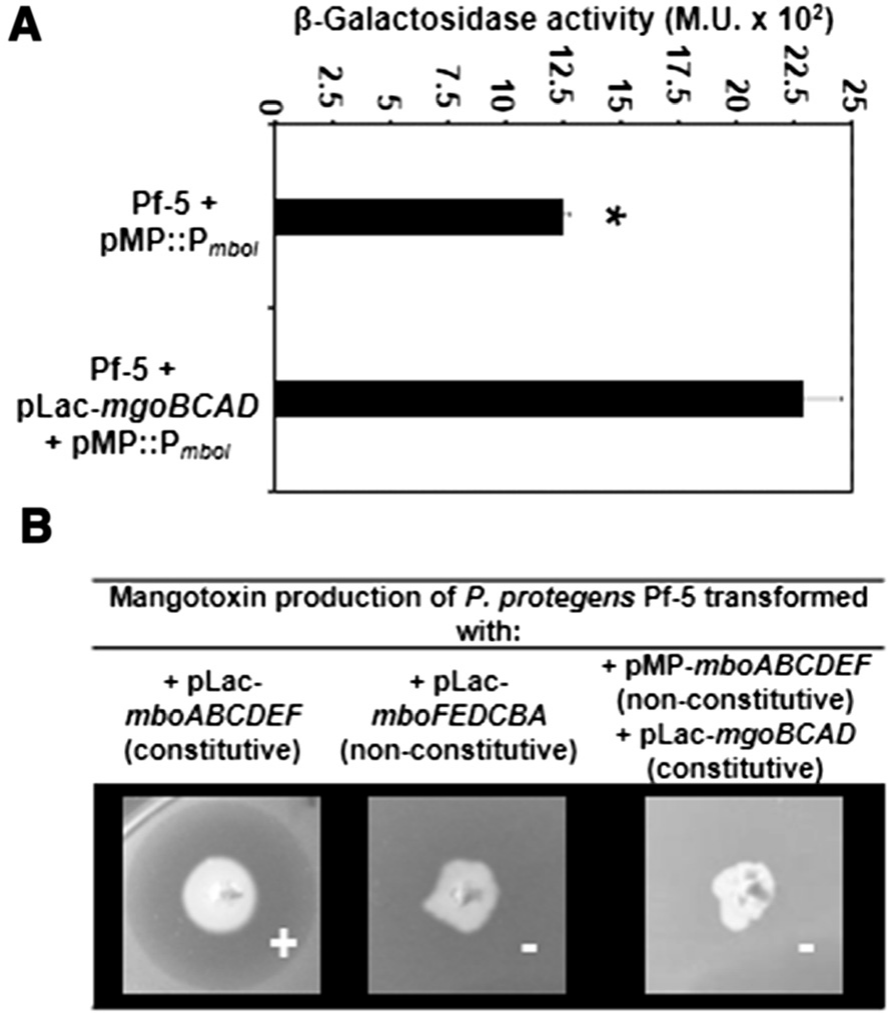
**Heterologous expression and production of mangotoxin. (A)** The *mbo* operon promoter activity in *P. protegens* Pf-5 transformed with the *mbo* operon promoter (pMP::P_*mboI*_) and with the empty promoter-probe vector pMP220 was used as a control. To check the positive regulation of the *mgo* operon, the strain Pf-5 was transformed with the vector pLac-*mgoBCAD*. The result is the average of three independent experiments performed in triplicate. Error bars indicate standard deviation. **(B)** Mangotoxin production of *P. protegens* Pf-5 transformed with pLac-*mboABCDEF* (*mbo* operon under its own and P_*LAC*_ promoter expression), pLac-*mboFEDCBA* (*mbo* operon under its own promoter expression) and pLac-*mgoBCAD* (*mgo* operon under its own and P_*LAC*_ promoter expression) and pMP220-*mboABCDEF* (*mbo* operon under its own promoter expression). Data were analysed for significance using a Student’s *t*-test (P = 0.05). Value of bar with an asterisk designation represent a statistically significant difference to the other bar value.

To further investigate if the *mgo* operon is able to regulate the expression of the *mbo* operon, we introduced the *mbo* operon promoter reporter construct (pMP::P_*mboI*_) and the *mgo* genes in *P. protegens* Pf-5, which lacks both the *mgo* and the *mbo* operons in its genome. Compared to the promoter activity in the wild-type Pf-5 background, a two-fold increase in ectopic *mbo* promoter activity was observed when Pf-5 was complemented with the *mgo* operon (Figure 4A). When *P. protegens* Pf-5 was transformed with pLac-*mboABCDEF* (*mbo* operon under pLac regulation), it produces mangotoxin. However, when *P. protegens* Pf-5 was transformed with pMP-*mboFEDCBA* (*mbo* operon under only its own promoter expression) it was not able to produce detectable amounts of mangotoxin, neither in absence nor in presence of the *mgo* operon of *P. syringae* pv. syringae UMAF0158 (Figure 4B). Therefore, the presence of the *mbo* and *mgo* operons in *P. protegens* Pf-5 would be not sufficient for the production of detectable amounts of mangotoxin.

## Discussion

The results of our study show that the regulation of mangotoxin biosynthesis in the plant pathogenic *P. syringae* pv. syringae strain UMAF0158 is governed by a complex interplay between the GacS/GacA two-component regulatory system, the nonribosomal peptide synthetase *mgoA* and the mangotoxin biosynthesis operon *mbo*. We showed that disruption of the *mbo* biosynthesis genes leads to reduced virulence. Introduction of the *mbo* operon in these biosynthesis mutants restored mangotoxin production but did not lead to full restoration of virulence on tomato leaflets. Multiple copies of the plasmid with the *mbo* operon could lead to overproduction of mangotoxin which may affect the regulation or production of other virulence factors such as syringomycin and syringopeptin.

**Figure 5:**
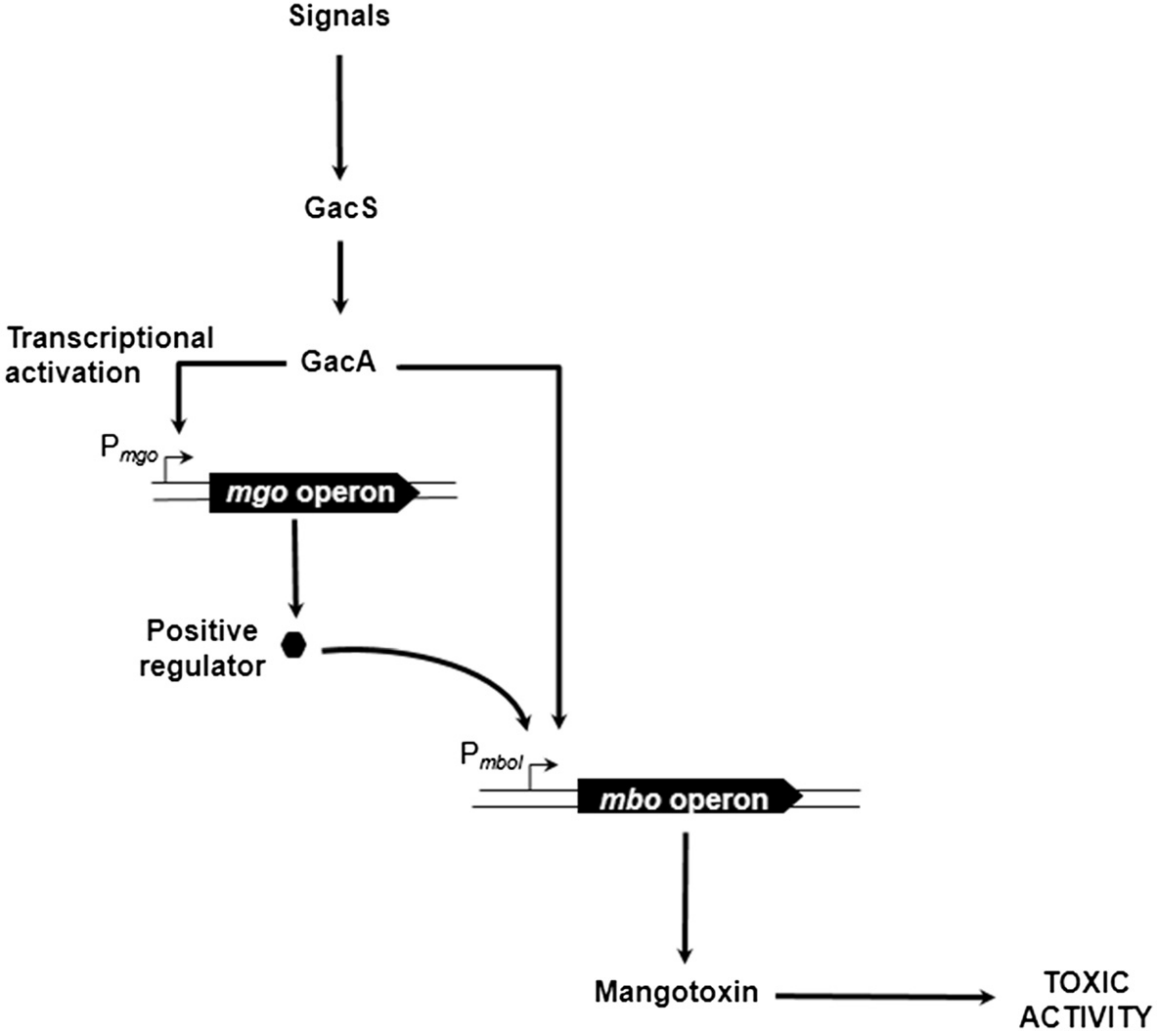
**Proposed model for regulation of mangotoxin biosynthesis in*P. syringae*pv. syringae.** In this model, GacS/GacA two-component regulatory system activates directly or indirectly the transcription of the *mgo* operon. And the *mgo* operon could synthetize a positive regulator of the *mbo* operon transcription. The *mbo* operon produces mangotoxin which acts as virulence factor.

Taken together the obtained results of this work and the previously described data [4,6,7], a simplified model for the interplay among these genes can be constructed (Figure 5). In this model, the GacS/GacA two-component regulatory system receives a yet unknown signal that activates a set of small RNAs [8,50,54]. The expression of genes regulated by the GacS/GacA might be mediated through the Rsm pathway [55,56]. In fact, components of this pathway such as the three small RNAs RsmX, RsmY and RsmZ and two RNA-binding proteins (RsmA and RsmE) were found in the genome of *P. syringae* pv. syringae UMAF0158 (Unpublished data). Transcriptional analysis of the *mgo*, *mbo* and *gac* genes showed that the *mbo* genes were markedly down-regulated in both the *gacA* and *mgoA* mutants. On the other hand, the transcriptional levels of *mgoB* and *mgoA*, also showed down-regulation in the *gacA* mutant, indicating that the *mgo* operon is also under regulation by the GacS/GacA two-component regulatory system. These data suggest that GacS/GacA is regulating the *mbo* operon expression via the *mgo* operon, however direct regulation of the *mbo* operon by the two-component regulatory system *gacS*/*gacA* cannot be excluded (Figure 5).

Transcriptional analysis with a *lacZ* fusion on the promoter of the *mbo* operon (P_*mboI*_), revealed that the product of the *mgo* operon could acts as positive regulator of *mbo* transcription. Interestingly, the *pvfC* gene (homologue of *mgoA*) is considered a regulator of virulence in *P. enthomophila,* but appears not to be part of the GacS/GacA regulatory cascade [28]. In strain UMAF0158, introduction of the *mgo* operon in a *gac* mutant could not restore mangotoxin production or *mbo*-promoter activity, suggesting that next to the *mgo* operon, additional factors are regulated by the *gac* system that influence mangotoxin production. It is worth noting that *P. entomophila* and *P. syringae* pv. syringae harbor two different genetic backgrounds, adapted to different environments. The first is found in diverse environments such as soil, aquatic ecosystems, rhizosphere, and in pathogenic interactions with *Drosophila melanogaster*[57]. The second is adapted for plant infection and epiphytic survival [3]. Therefore, the regulatory roles of these orthologues can substantially differ between these two *Pseudomonas* species. On the other hand, the fact that both PvfC and MgoA are involved in the regulation of virulence could indicate that in other *Pseudomonas* spp. these factors would be involved in the regulation of virulence and/or secondary metabolite production.

Phylogenetic analysis of MgoA and the adenylation domains suggested an evolutionary specialization of this protein into the *Pseudomonas* genus. In this context, it is worth noting that the transformation of the *mbo* operon under the expression of its own promoter only confers mangotoxin production in the *P. syringae* group and not in the *P. fluorescens* group. Therefore, it seems that the NRPS MgoA is involved in different signal transduction pathways depending of the *Pseudomonas* species. In the case of *P. syringae*, MgoA appears to activate mangotoxin production. It remains to be studied if MgoA is also involved in the regulation and production of other antimetabolites in the *P. syringae* group, such as tabtoxin and phaseolotoxin. The positive regulation of the *mbo* operon promoter activity in the presence of the *mgo* operon in Pf-5, combined with the lack of detectable amounts of mangotoxin suggests that additional factors for mangotoxin biosynthesis or its export are not present in the *P. fluorescens* group.

## Conclusions

In summary, for *P. syringae* pv. syringae UMAF0158, the GacS/GacA two-component system regulates transcription of the *mgo* and *mbo* operons and thereby mangotoxin biosynthesis. At the same time, the *mgo* operon product seems to act as a positive regulator of the *mbo* operon. The proposed model for mangotoxin biosynthesis is a simplified and initial overview of the interaction between the *gac*, *mgo* and *mbo* gene products based on the results obtained in the current study. This is the first evidence of the interplay between MgoA and the GacS/GacA two-component regulatory system in the regulation of the mangotoxin biosynthesis.

### Ethics statement

We the authors hereby declare that the research performed with plants has been conducted in accordance with institutional, national and international guidelines.

## Competing interests

We the authors hereby declare that there is no conflict of interests concerning this manuscript.

## Authors’ contributions

VJC, MV, EA, AV, JMR and FMC conceived the study. VJC and EA did all the cloning and genetics of this study. VJC and MV did the Q-PCR experiments and analysis. VJC and JAG did complementation and reporter construct experiments. JMR and AV supported the research. VJC, MV, JMR and FMC wrote the manuscript. VJC, EA, MV, AV, JMR and FMC coordinated and critically revised the manuscript. All authors read and approved the manuscript.

## Supplementary Material

Additional file 1: Table S1 Primers used in this study. Click here for file

Additional file 2: Figure S1 Growth characteristics of *P. syringae* pv. syringae strain UMAF0158 and the derivatives *mgoA* and *gacA* mutants. (A) Growth of the wild type strain UMAF0158 and the *mgoA* (∆*mgoA*) and *gacA* (2βB7) mutants at 22ºC in PMS. At each time point, the bacterial density was estimated by serial dilutions and colony counts on plates of selective medium and expressed as log cfu ml^-1^ of culture. (B) UMAF0158 mangotoxin production at 22ºC in PMS. At each time point, the mangotoxin production was estimated using cell-free filtrate and represented as the previously defined toxic units (T.U.). The dashed line represents the detection limit of the technique. Mean values for three replicates are given; the error bars represent the standard errors of the mean. Click here for file

Additional file 3: Figure S2 Virulence analysis of the wild type strain *P. syringae* pv. syringae UMAF0158 and corresponding derivatives using a detached tomato leaf assay. (A) *In planta* growth inside the tomato leaflets after H_2_O_2_ surface disinfection of the wild type strain UMAF0158, *mgoA* and *mboA* mutants, and their respective complemented derivatives. (B) Severity of necrotic symptoms (necrotic area) on tomato leaflets inoculated with wild type strain UMAF0158, the mutants in *mboA* and *mgoA* with their respective complemented derivatives. The total necrotic area (mm^2^) from 30 inoculated points on tomato leaflets was measured 10 days after inoculation and used to compare the severity of necrotic symptoms produced by the different strains. (C) Representative pictures of the necrotic lesions produced by the wild type strain and the different mutants at 10 dpi. Different letters denote statistically significant differences at *p =* 0.05, according to analysis of variance followed by Fisher’s least significant difference test. Click here for file

Additional file 4: Figure S3 *mboACE* transcript levels in the wild type strain UMAF0158. Relative expression of the genes involved in the mangotoxin biosynthesis at the different time points during the growth curve. For each time point, mean values of four biological replicates are given; the error bars represent the standard errors of the mean. Click here for file

Additional file 5: Figure S4 Phylogenetic analysis of the MgoA of different *Pseudomonas* spp. Neighbor-joining tree was constructed with MEGA5 using a partial sequence of MgoA. The boxes indicate the different groups of *Pseudomonas* and the presence (*mbo* +) or absence (*mbo* -) of the *mbo* operon. The evolutionary history was inferred using the Neighbor-Joining method [52]. The evolutionary distances were computed using the JTT matrix-based method [53] and are in the units of the number of amino acid substitutions per site. The rate variation among sites was modelled with a gamma distribution. The analysis involved 126 amino acid sequences. There were a total of 1015 positions in the final dataset. Evolutionary analyses were conducted in MEGA5 [45]. *Burkholderia cenocepacia* J2315 was used as the outgroup. Bootstrap values (1,000 repetitions) are shown on the branches. Click here for file
