# Supplementary material for: Mangotoxin production of Pseudomonas syringae pv. syringae is regulated by MgoA
Source: BMC Microbiol. 2014 Feb 21;14:46. doi: 10.1186/1471-2180-14-46 (PMC3945005; doi:10.1186/1471-2180-14-46)
Supplement: Additional file 1: Table S1 — Primers used in this study. [file 1471-2180-14-46-S1.doc]

**Additional file 5: Table S1** **Primers used in this study.**

| **Fragment** | **Orientation** | **Primer sequence^a^** |
| --- | --- | --- |
| ***Transcriptional analysis*** |  |  |
| *mboA* | Forward | 5´-TTCACATGAACAACGAACTG-3´ |
|  | Reverse | 5´-GCCGAACTCATCATACAAAT-3´ |
| *mboC* | Forward | 5´-TCAATGAGTTGGGAAACATC-3´ |
|  | Reverse | 5´-TGTTGCGCACTTTCTGTACT-3´ |
| *mboE* | Forward | 5´-GAGTTGGCCGAAATCATC-3´ |
|  | Reverse | 5´-TCCGGTAGCTCTCGTAGGTA-3´ |
| *mgoA* | Forward | 5´-CAACCAGACGCTCAGCTAT-3´ |
|  | Reverse | 5´-CTCAGGCAGATACCAATCAC-3´ |
| *mgoB* | Forward | 5´-GCCTGATCAACGAGATTGT-3´ |
|  | Reverse | 5´-GTCTCTGCAGTTCGATGAAG-3´ |
| *rpoD* | Forward | 5´-GATGCCTTCTTCGATACGTT-3´ |
|  | Reverse | 5´-ACCGATCCTGTTCGTATGTA-3´ |
| ***Complementation*** |  |  |
| *mgo* operon | Forward | 5´-CAAATCTAGAACCAAGGCCAAGTCGACC-3´ |
|  | Reverse | 5´-CCCTCTAGAGAGTAGGTCATCGTCAAG-3´ |
| *mbo* operon | Forward | 5′-AGGCGAATTCGCGCATAGCGATCG-3′ |
|  | Reverse | 5′-CGCCTGCAGGACCAGCACCACCAG-3′ |

^a^ The 5´ end of the forward and reverse primers contain the restriction sites (underlined) for *Xba*I which is required for cloning into pBBR1MCS-5 and *EcoR*I and *Pst*I for cloning in the pMP220.
