# Supplementary figures and images for: Mangotoxin production of Pseudomonas syringae pv. syringae is regulated by MgoA
Source: BMC Microbiol. 2014 Feb 21;14:46. doi: 10.1186/1471-2180-14-46 (PMC3945005; doi:10.1186/1471-2180-14-46)

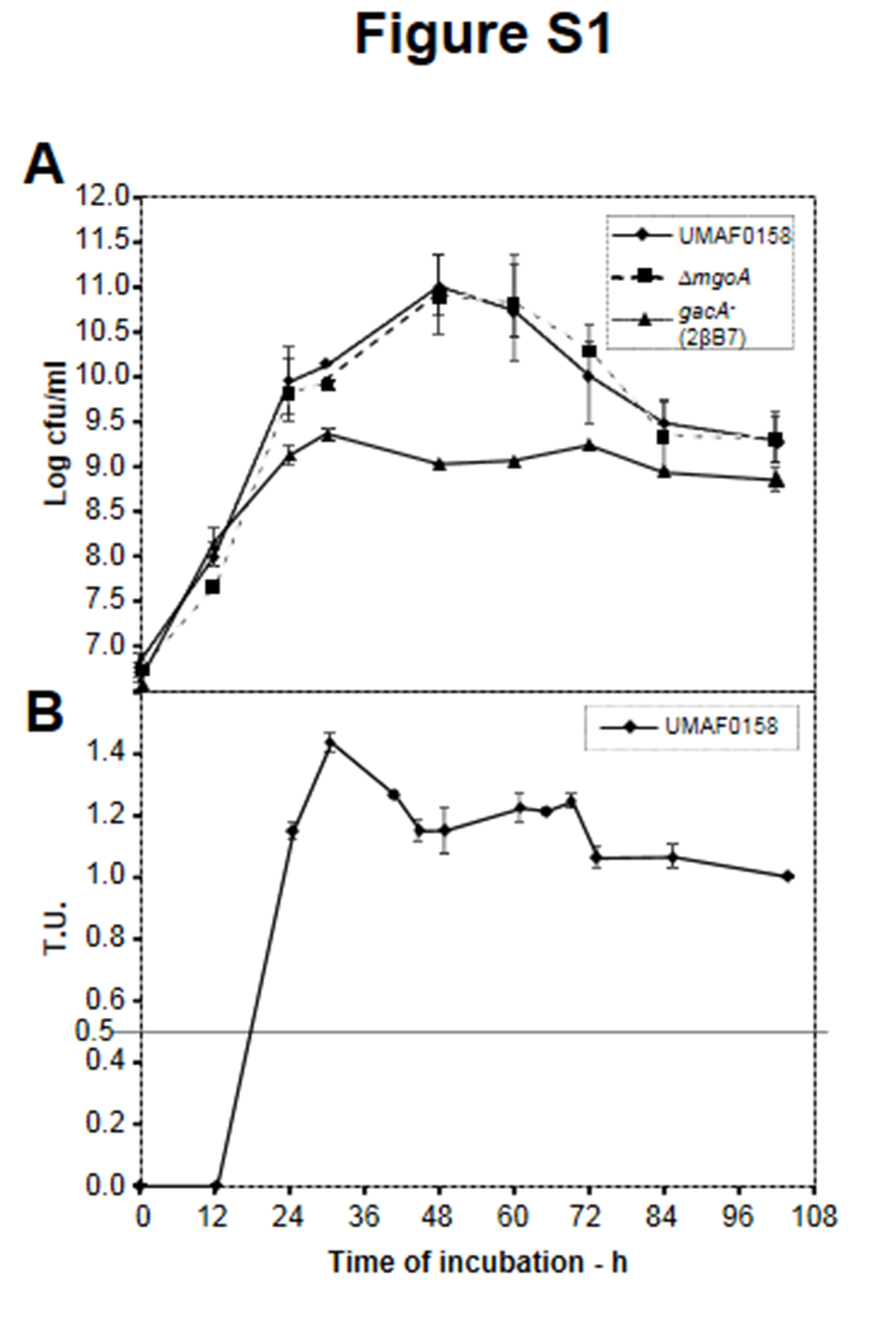

Supplement: Additional file 2: Figure S1 — Growth characteristics of P. syringae pv. syringae strain UMAF0158 and the derivatives mgoA and gacA mutants. (A) Growth of the wild type strain UMAF0158 and the mgoA (∆mgoA) and gacA (2βB7) mutants at 22ºC in PMS. At each time point, the bacterial density was estimated by serial dilutions and colony counts on plates of selective medium and expressed as log cfu ml-1 of culture. (B) UMAF0158 mangotoxin production at 22ºC in PMS. At each time point, the mangotoxin production was estimated using cell-free filtrate and represented as the previously defined toxic units (T.U.). The dashed line represents the detection limit of the technique. Mean values for three replicates are given; the error bars represent the standard errors of the mean. [file 1471-2180-14-46-S2.tiff]

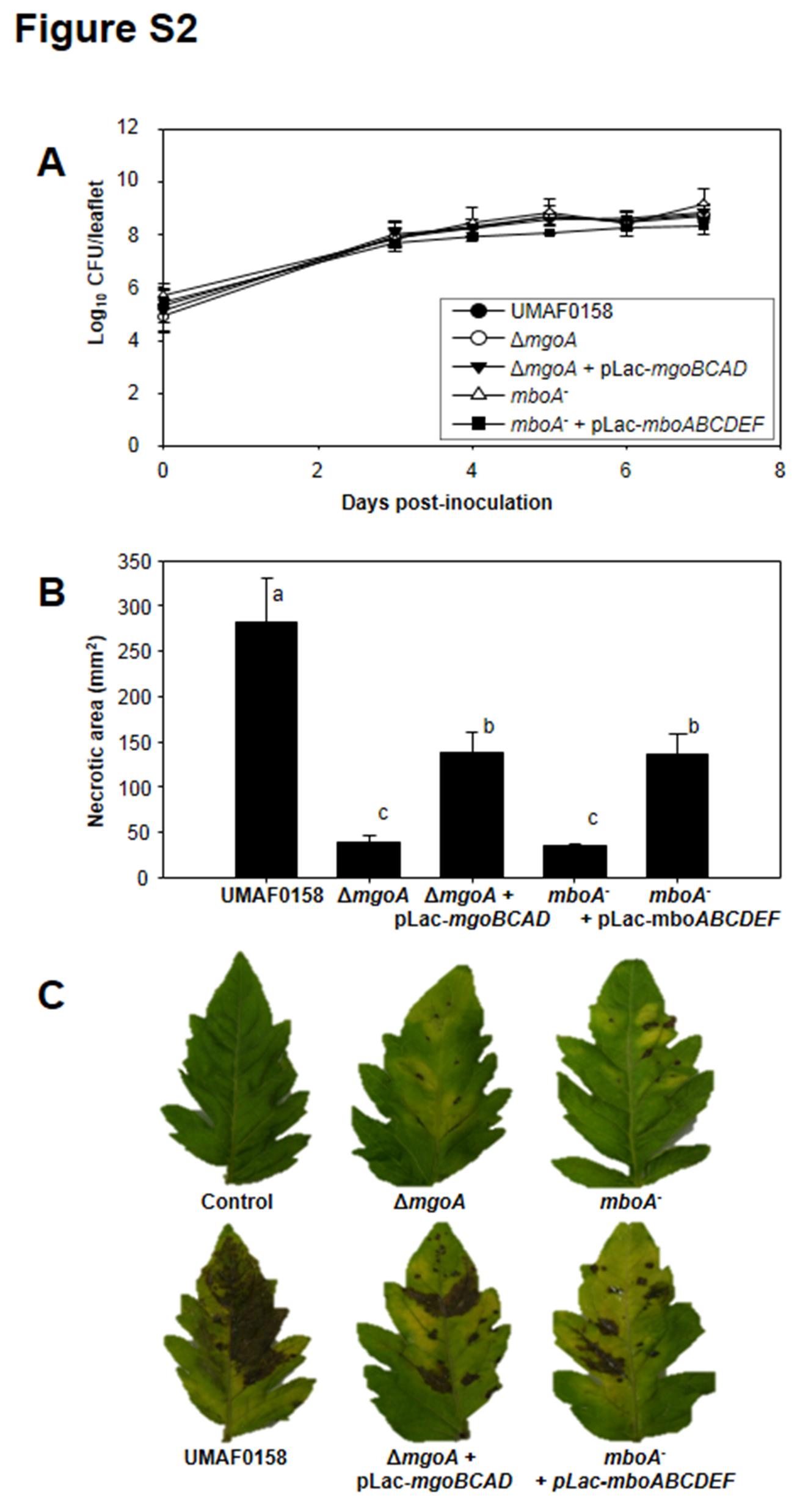

Supplement: Additional file 3: Figure S2 — Virulence analysis of the wild type strain P. syringae pv. syringae UMAF0158 and corresponding derivatives using a detached tomato leaf assay. (A) In planta growth inside the tomato leaflets after H2O2 surface disinfection of the wild type strain UMAF0158, mgoA and mboA mutants, and their respective complemented derivatives. (B) Severity of necrotic symptoms (necrotic area) on tomato leaflets inoculated with wild type strain UMAF0158, the mutants in mboA and mgoA with their respective complemented derivatives. The total necrotic area (mm2) from 30 inoculated points on tomato leaflets was measured 10 days after inoculation and used to compare the severity of necrotic symptoms produced by the different strains. (C) Representative pictures of the necrotic lesions produced by the wild type strain and the different mutants at 10 dpi. Different letters denote statistically significant differences at p = 0.05, according to analysis of variance followed by Fisher’s least significant difference test. [file 1471-2180-14-46-S3.tiff]

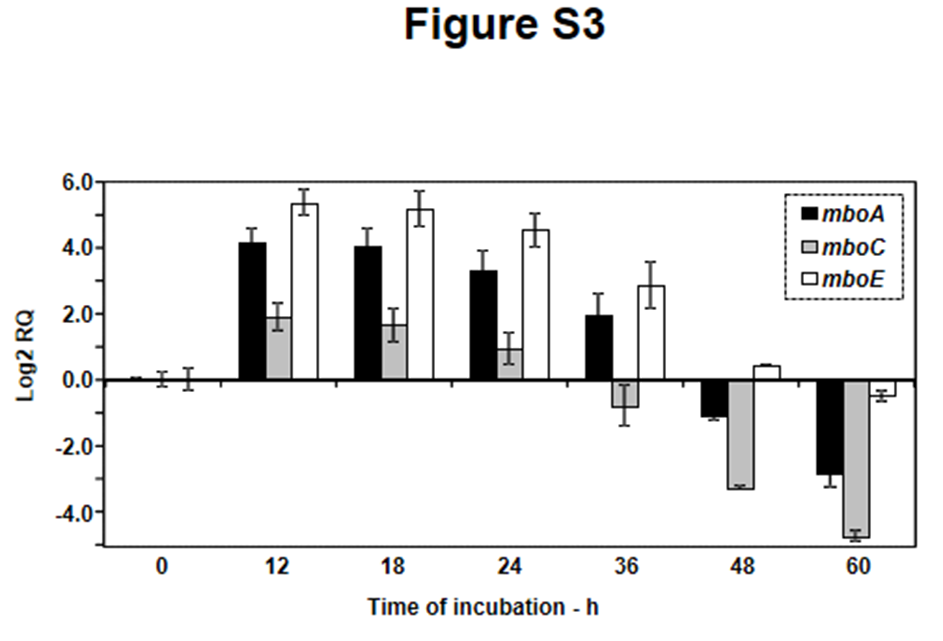

Supplement: Additional file 4: Figure S3 — mboACE transcript levels in the wild type strain UMAF0158. Relative expression of the genes involved in the mangotoxin biosynthesis at the different time points during the growth curve. For each time point, mean values of four biological replicates are given; the error bars represent the standard errors of the mean. [file 1471-2180-14-46-S4.tiff]

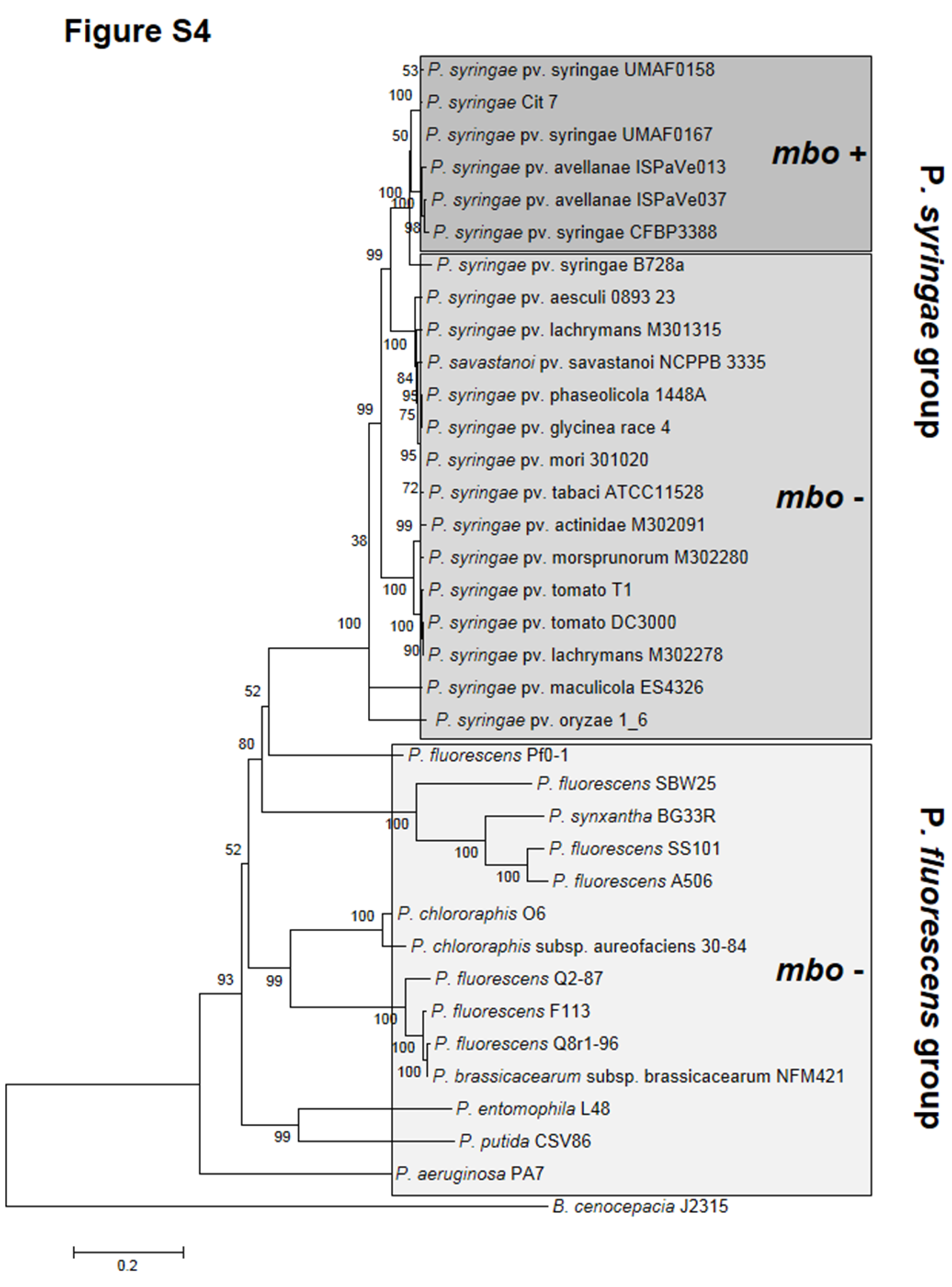

Supplement: Additional file 5: Figure S4 — Phylogenetic analysis of the MgoA of different Pseudomonas spp. Neighbor-joining tree was constructed with MEGA5 using a partial sequence of MgoA. The boxes indicate the different groups of Pseudomonas and the presence (mbo +) or absence (mbo -) of the mbo operon. The evolutionary history was inferred using the Neighbor-Joining method [52]. The evolutionary distances were computed using the JTT matrix-based method [53] and are in the units of the number of amino acid substitutions per site. The rate variation among sites was modelled with a gamma distribution. The analysis involved 126 amino acid sequences. There were a total of 1015 positions in the final dataset. Evolutionary analyses were conducted in MEGA5 [45]. Burkholderia cenocepacia J2315 was used as the outgroup. Bootstrap values (1,000 repetitions) are shown on the branches. [file 1471-2180-14-46-S5.tiff]
